# Death Unpreparedness Due to the COVID-19 Pandemic: A Concept Analysis

**DOI:** 10.3390/healthcare12020188

**Published:** 2024-01-12

**Authors:** Cristina Costeira, Maria Anjos Dixe, Ana Querido, Ana Rocha, Joel Vitorino, Cátia Santos, Carlos Laranjeira

**Affiliations:** 1 Centre for Innovative Care and Health Technology (ciTechCare), Rua de Santo André-66-68, Campus 5, 13 Polytechnic University of Leiria, 2410-541 Leiria, Portugal; maria.dixe@ipleiria.pt (M.A.D.); ana.querido@ipleiria.pt (A.Q.); catia.santos@ipleiria.pt (C.S.); 2 School of Health Sciences, Polytechnic University of Leiria, Campus 2—Morro do Lena, Alto do Vieiro—Apart. 4137, 2411-901 Leiria, Portugal; joel.vitorino@ipleiria.pt; 3 The Health Sciences Research Unit: Nursing (UICISA:E), Nursing School of Coimbra (ESEnfC), 3004-011 Coimbra, Portugal; 4 Center for Health Technology and Services Research (CINTESIS), NursID, University of Porto, 4200-450 Porto, Portugal; 5 Nursing School of Coimbra, Avenida Bissaya Barreto s/n, 3004-011 Coimbra, Portugal; anamnrocha@esenfc.pt; 6 Palliative Care Unit, Portuguese Institute of Oncology of Coimbra, 3000-075 Coimbra, Portugal; 7 Comprehensive Health Research Centre (CHRC), University of Évora, 7000-801 Évora, Portugal

**Keywords:** preparedness for death, COVID-19, end of life, concept analysis

## Abstract

The COVID-19 pandemic imposed changes upon the capacity of healthcare systems, with significant repercussions on healthcare provision, particularly at end-of-life. This study aims to analyze the concept map of death unpreparedness due to the COVID-19 pandemic, capturing the relationships among the attributes, antecedents, consequences, and empirical indicators. Walker and Avant’s method was used to guide an analysis of this concept. A literature search was performed systematically, between May 2022 and August 2023, using the following electronic databases on the Elton Bryson Stephens Company (EBSCO) host platform: Medical Literature Analysis and Retrieval System Online (Medline), Psychological Information Database (PsycINFO), Cumulative Index to Nursing and Allied Health Literature (CINAHL) Complete, Cochrane Library, and Nursing and Allied Health Collection. Thirty-four articles were retrieved. The unexpected and unpredictable impositions associated with inexperience and unskillfulness in dealing with COVID-19 configured challenges for healthcare professionals, family/caregivers, and even the dying person. Nine key attributes emerged in three main domains: (1) Individual: (a) disease-related conditions, (b) separation distress, and (c) scarcity of death and grief literacy; (2) Relational: (a) Dying alone, (b) poor communication, and (c) existential issues; and (3) Contextual: (a) disrupted collective mourning and grieving, (b) disrupted compassionate care and, (c) pandemic social stigma. This study contributed a full definition of death unpreparedness in a global pandemic scenario such as COVID-19. In this sense, feeling unprepared or unready for death brought new challenges to the bioecological resources of those affected. It is essential to embrace strategies capable of providing emotional and spiritual support in the dying process and to respect patient wishes. The lessons learned from COVID-19 should be applied to events with a comparable impact to minimize their consequences.

## 1. Introduction

The COVID-19 disease brought several changes to daily life, even within the death process. The sum of deaths reported by the World Health Organization [WHO] was exceedingly high, reaching 6,956,900 deaths by 9 September 2023 [1,2].

This period was characterized by sudden and rapid changes in the process of dying and grieving, not only for people who were dying but also for their families, informal caregivers, and even Healthcare Professionals (HCPs) [3,4]. This global fact changed how populations lived and experienced the death process. Changes in the places of death, the situations of solitary dying created by the sanitary measures, and the impossibility of saying goodbye and performing traditional funeral rites hindered the mourning and grief process [5,6,7]. According to Worden [8], the grieving process involves four grief tasks necessary for a critical, irrepressible, and singular adaptation to the new reality: (i) accepting the reality of the loss; (ii) processing the pain of grief; (iii) adjusting to a world without the dead person; and (iv) finding a way to remember the dead person while embarking on the rest of one’s journey through life. Contrary to an individual perspective of the grieving process, Worden’s approach defends that this process implies the mourner’s active investment and that time by itself is not enough for integrating grief. More recently, grief interventions defend an integrative approach based on individual models of grief, grief tasks, crisis coping, and family theories of interaction applied to grief and loss. These should consider several specific aspects of all those involved and identify factors related to the two major sources of distress: traumatic and relational aspects of end-of-life (EoL) caregiving [9,10,11,12,13].

With COVID-19, the possibility of solving the grief tasks and experiencing death suffered drastic changes. The uncertainty and suddenness of the new reality implied difficulties in finishing life’s tasks, solving issues with family and friends, and making peace with the inevitable, thus making it difficult to be prepared for death [14,15].

Death preparedness is a dynamic and complex concept. Incorporating information and attitudes into an integrated awareness and acceptance of one’s impending death is required for cognitive, emotional, and practical preparation for death [16,17]. Emotional preparedness for death implies emotionally embracing one’s dying role, being realistic about one’s current circumstances, giving up on unlikely scenarios of survival, and closing, reconciling, and repairing interpersonal links with loved ones to prepare them for life without oneself [18]. Numerous research studies have looked at the links between patients’ psychological well-being, quality of life at the EoL, and preparedness for death [19,20]. Evidence also suggests that death preparedness fosters a good quality of care [21].

Two operationalizations of preparedness are distinguished in the literature: death preparedness and caregiving preparedness. While “preparedness for caregiving” pertains to a patient’s level of readiness to undertake caregiving responsibilities, “preparedness for death” concerns the readiness of a loved one’s family members to face their demise [22]. Although research has been conducted on both constructs, evaluating, discussing, and promoting death preparedness remains a difficult task for policymakers, scholars, and HCPs.

High levels of pre-loss grief promoted poor post-loss adaptation by caregivers of cancer patients [23]. Conversely, high levels of preparedness were a protective factor against poor adjustment to death. Since these factors influence postmortem adjustment, it is crucial to examine the predictors of death preparedness and pre-loss grief in order to gain insight into the bereavement process and develop specialized interventions to stabilize death preparedness and pre-loss grief [22]. The pandemic scenario highlighted these concerns. While many studies presume that COVID-19 had a detrimental influence on the bereavement process [24], few studies evaluated the effect of COVID-19 on death preparedness. Grieving during the pandemic became disorganized because COVID-19 deaths were more unexpected and traumatic than other types of deaths [25].

### Research Problem

The sudden nature of the COVID-19 pandemic altered the processes of death and death preparedness, preventing the socialization of death, and determining, in many cases, a high risk of traumatic grief for surviving family members [26,27].

The concept needs to be clarified because the unpreparedness for COVID-19 deaths has conceptual, theoretical, and practical ambiguities and complexities. The purpose of this concept analysis was to develop and clarify the definition of death unpreparedness due to COVID-19 by describing the connections to related principles and suggesting future implications for practice, research, and education in similar critical events. 

In addition to the severity of the situation, the high mortality resulting from the lack of preparedness during the COVID-19 pandemic teaches us numerous valuable lessons. With the current state of globalization and interconnectedness, COVID-19 was not the first and will not be the last pandemic that humanity must face. During the COVID-19 pandemic, significant progress in science and medicine allowed us to detect and manage diseases in ways that earlier pandemics could not have conceived. Stakeholders must exhibit agility and responsiveness to changes. Therefore, an in-depth clarification of death unpreparedness experienced during COVID-19, from the perspective of patients, family caregivers, and professionals, will contribute to learning from experience and better preventing the phenomenon. In this regard, our main research question is, “What antecedents, attributes, and consequents define the death unpreparedness concept in a global pandemic scenario like COVID-19?” 

## 2. Materials and Methods

### 2.1. Study Design 

A concept analysis using Walker and Avant’s [28] strategic method was used to define and elucidate the phenomenon of unpreparedness for death during the COVID-19 pandemic. Those authors opined that concept analysis is a scientific investigation of a concept to find out its meaning and misconceptions. 

Walker and Avant [28] (p. 8) proposed eight iterative steps involved in concept analysis: “(1) the selection of a concept; (2) the determination of the analysis purpose; (3) the identification of all possible uses of the concept; (4) the creation of the defining attributes; (5) identification of a model case of the concept; (6) identification of borderline and contrary cases; (7) identification of antecedents and consequences; and, (8) the definition of empirical referents”. 

This method was chosen because it allows an iterative analysis that helps to remediate the lack of consensus over the concept’s key attributes [28,29]. Although the model’s stages imply they are sequential, the authors [28] suggest they are iterative, which means revisiting or returning to former steps as data emerges.

### 2.2. Study Details

In this concept analysis, we reviewed the relevant published literature. The database research took place between May 2022 and September 2022 and was updated in August 2023. The search for relevant articles was guided by an expert librarian using the following databases through the Elton Bryson Stephens Company (EBSCO) host platform: Medical Literature Analysis and Retrieval System Online (Medline), Psychological Information Database (PsycINFO), Cumulative Index to Nursing and Allied Health Literature (CINAHL) Complete, Cochrane Library, and Nursing and Allied Health Collection. The terms were searched individually using the Boolean term OR and then combined using the Boolean term AND. In some instances, truncation, denoted by an asterisk (*), was used to include numerous suffixes within the retrieved search result. Articles were incorporated into the initial screening if the search term was present in both the title and abstract. The search method was derived from the Medline database and applied to additional databases. The following search string was used with a combination of free text and controlled vocabulary: ((“COVID-19” [MeSH term] OR “COVID-19” [Title/Abstract] OR “SARS-CoV-2” [Title/Abstract] OR “Coronavirus Disease-19” [Title/Abstract] OR “COVID-19 Pandemics” [Title/Abstract]) AND (“Death” [MeSH term] OR “Death*” [Title/Abstract] OR “Dying Process” [Title/Abstract] OR “Dying Care” [Title/Abstract] OR “Grief” [MeSH term] “Grief” [Title/Abstract] OR “Bereavement” [MeSH term] OR “Bereave*” [Title/Abstract] OR “Personal Loss” [Title/Abstract] OR “Preparedness of death” [Title/Abstract] OR “End-of-Life” [Title/Abstract] OR “mourn*” [Title/Abstract] OR “Attitude to Death” [MeSH term] OR “Attitude to Death” [Title/Abstract])). 

In the first step, all articles collected were verified to see if they were duplicated and removed in those cases. Furthermore, two independent reviewers (C.C and C.S.) conducted a screening of titles and abstracts to evaluate their suitability based on the inclusion criteria established for the review. The potentially relevant articles were obtained in full text. They were then distributed between four reviewers (A.Q., M.A.D., A.R., and J.V.) who thoroughly evaluated the whole text following the inclusion criteria. In instances where disagreements emerged amongst the reviewers throughout each phase of the selection process, a resolution was sought via the involvement of a third reviewer (C.L.). The references of the identified papers were further examined to locate relevant research.

Articles found during the search were added to reference management software (Mendeley, Elsevier, Amsterdam, The Netherlands, v2.107.0). Data were organized in an Excel spreadsheet that included publication type, study location, design, aims, participants, and key findings. A qualitative thematic analysis [30], as an iterative process of coding, was used to identify and delineate antecedents, attributes, and consequences. This was an ongoing process of systematically arranging and rearranging data until a complete and relevant set of descriptors was created. This option was useful because the concept under analysis was expected to be unclear in the available literature, given the sudden pandemic reality. Using a synthesis matrix, we summarized the main themes and then categorized them according to the concept analysis. 

Since this is not a systematic review, we did not assess the methodological quality or risk of bias of the included articles, in line with Walker and Avant’s concept analysis method [28].

### 2.3. Eligibility Criteria

The inclusion criteria consisted of the following: (1) peer-reviewed articles in English, Portuguese, or Spanish languages; (2) any primary study design (quantitative, qualitative, and mixed-method studies) or secondary study (literature reviews and narrative reviews) containing information about the concept of being unprepared for death due to the COVID-19 pandemic; (3) involved one or more representatives of dying people, families, and health professionals; and (4) published in the last three years, between December 2019 and May 2023 (the World Health Organization announced the end of the global pandemic on 5 May 2023). 

Exclusion criteria included the gray literature (books, theses, and dissertations), letters to the editor, conference proceedings, and opinion papers.

## 3. Results

**Figure 1:**
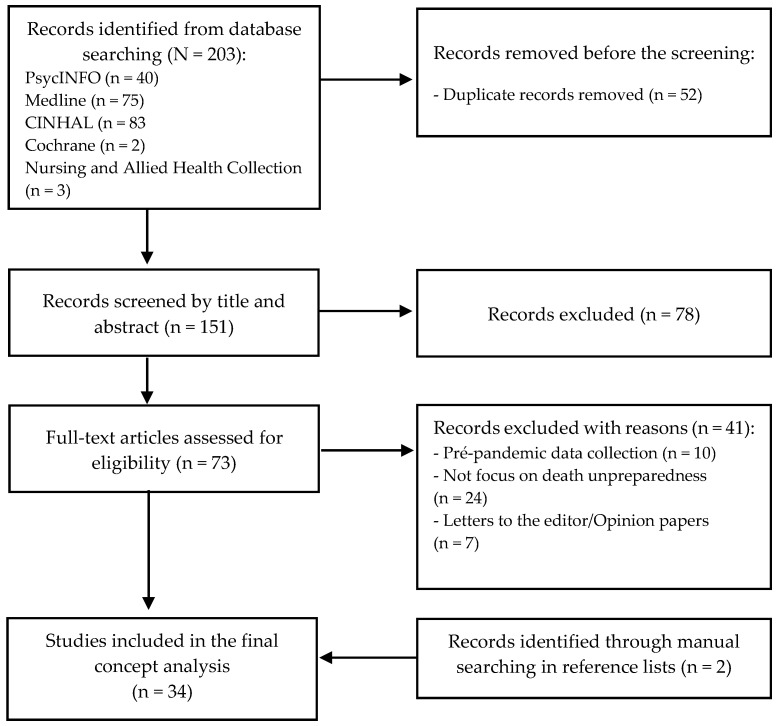
Flow diagram illustrating selection of sources.

The initial search retrieved 203 articles. After removing the 52 duplicates, 151 articles remained to be reviewed by title and abstract. This review excluded 78 articles. A full review of 73 articles was performed. Among these, 41 were excluded because of the type of study (letters to the editor and opinion papers), no focus on the topic, or the data collection before COVID-19, resulting in 32 articles. Two additional articles were retrieved by searching the article bibliographies. In the end, a total of 34 articles were included in the concept analysis. The results of the search are shown in a flow diagram (see Figure 1). 

Among the retrieved studies, 44.1% were quantitative research, 41.2% were qualitative research, 8.8% were mixed-methods studies, and 5.9% were narrative reviews. Most of the studies (55.9%) were developed in European countries and were focused on bereaved relatives (68.8%). Lastly, 61.8% of the articles were published in 2021, 26.5% were published in 2022, 8.8% were published in 2020, and 2.9% were published in 2023. An overview of included sources is provided in Supplementary Table S1. 

### 3.1. Definitions and Uses of the Concept

The adjective “unprepared” implies being unready or lacking a plan for some activity, purpose, or event. Relatedly, the term “unpreparedness” is defined as the lack of preparation to face expected responsibilities [31].

The encounter with death and the accompanying phase of grieving are significant sources of stress and change in an individual and family’s life trajectory. It can be effectively managed by proactive planning [19]. However, some catastrophic events can disrupt this possibility and generate an unexpected death, defined as death that occurs suddenly and sooner than expected. An abrupt event resulting in untimely death is accompanied by an element of surprise that implies a lack of knowledge and skill to face the new situation (inexperience and unskillfulness in dealing with death). Unexpected deaths are frequently catastrophic events that have significant socioemotional consequences for all. Because the assessment of unexpected events can only be made retrospectively (rather than prospectively), the dying person, families, and HCPs are unprepared to face death and the dying process. Lastly, because these events are unpredictable, the etiology is frequently unknown and its course is poorly documented, making this topic more difficult to investigate. Any sort of sudden and/or traumatic death presents significant challenges to the emotional, physical, and spiritual resources of bereaved people.

### 3.2. Attributes

According to the Walker and Avant Model [28], the process of defining attributes is “the heart” of concept analysis. The extended literature analysis revealed the most common terms associated with death unpreparedness from the perspective of the dying person, family caregivers, and HCPs. 

Nine key attributes emerged from the literature, experienced in three domains (see Table 1):

(1) Individual domain: emerging as a phenomenological experience of the dying person and their families, including the attributes, (a) disease-related factors, (b) separation distress, and (c) paucity of death and grief literacy;
(2) Relational domain: referring to (a) dying alone, the intrapersonal experience, (b) poor interpersonal communication and the interpersonal experience, and (c) transcendence facing existential issues experienced by the dying person, family, and healthcare professionals;
(3) Contextual domain: encompassing the care, rules, and regulations contributing to death unpreparedness such as (a) disrupted collective mourning and grieving, (b) disrupted compassionate care, and (c) the pandemic’s social stigma.

### 3.3. Antecedents

The antecedents comprise the events that precede the concept of interest [28]. Based on the antecedents, one can identify the different contexts in which a definition may be used. The present research synthesized antecedents related to the concept of preparedness for death during COVID-19 into individual and collective domains. 

The individual domains that precede the preparedness for death during COVID-19, and interfere with how people construct the concept, are different depending on gender [33], age [58], and specific cultural, spiritual, and religious beliefs that can help develop resilience in stressful situations related to health and grief [34,40,49,59]. Other events that precede death preparedness and can induce an absence of spiritual well-being [35] include bereavement by a friend’s death [43], negative death attitudes from previous experiences [35,42,46,55,60], and death confrontation.

The concept is preceded by contextual domains: the place of death [35,47,51,59]; quarantine measures, such as social distancing policies to control contagion and travel prohibitions [32,34,36,39,40,47,48,50,60,61]; unpredictability, the unexpected increase in death numbers [33,60]; and lockdowns on contacts and visits [47,48,57,60], which impede the resolution of previous conflicts [33]. When death occurred, the impossibility of funeral ceremonies or their postponement until 6 months after death [60] caused family suffering due to religious and cultural beliefs [34]. 

Changes in a professional context interfered with non-preparedness for death during COVID-19, such as unpredictability [62], broader changes [54] in the workplace and teamwork associated with the rapid opening of new services, changes in prescription patterns, changes in working hours, inconsistent training, changes in human and care resources, and a lack of support for patients and families in the community [62].

### 3.4. Consequences

Significant strains are placed on the bereaved or healthcare professional’s emotional, physical, and spiritual resources in the event of a sudden death caused by COVID-19. Thus, death unpreparedness due to COVID-19 has numerous repercussions for the bereaved family and frontline HCPs (see Table 2). 

Approaching their EoL, most individuals do not make provisions for the type of health care they would prefer; consequently, they have a diminished chance of experiencing the type of demise they envision. An individual whose demise was anticipated to take several months of gradual deterioration may ultimately pass away unexpectedly. They have been expected to deteriorate progressively, yet, remain healthy until their untimely demise. Those near such fatalities may not perceive them as sudden at all, especially if the deceased was suffering. Notably, preparing to provide care for family members who are gravely ill entails developing an understanding of death, safeguarding against adverse health outcomes, and reducing the burden of bereavement.

### 3.5. Empirical Referents

Without a standardized measurement instrument, death unpreparedness is a difficult concept to evaluate, according to the reviewed literature. In the pre-pandemic world, an instrument composed of a single item was used to capture death preparedness. Respondents were asked, “If your loved one were to die soon, how prepared would you be for his/her death?”. Response options were “not at all,” “somewhat,” and “very” [16,17]. This instrument’s utility has not been validated during the pandemic period. To mitigate this issue, additional novel tools should be developed, specifically those that adopt a multidimensional perspective encompassing the physical, psychological, familial, social, spiritual, and cultural aspects of death unpreparedness in the face of abrupt illnesses and unforeseen circumstances.

In parallel, other instruments capture concepts that may have some proximity to our core concept, namely: (1) The Pandemic Grief Scale (PGS), composed of five items, evaluates the presence of problematic grief symptoms in individuals who have experienced the loss of a loved one during the COVID-19 pandemic [43,57]; (2) The Traumatic Grief Inventory Self Report Plus (TGI-SR+) measures grief severity [66]. This 22-item scale evaluates current criteria for Prolonged Grief Disorder as per DSM-5-TR and ICD-11 [36]; (3) The ‘Care of the Dying Evaluation’ (CODE™) questionnaire focuses on quality of care and family support during the last days of life [55]; and (4) The Work and Social Adjustment Scale (WSAS) assesses functional impairment experienced due to a COVID-19 loss [57]. 

### 3.6. Case Examples

Walker and Avant’s approach [28] demands the identification of a case model and borderline and contrary cases. Although we used real-life examples, ethical approval was not required since this study did not obtain private identifiable information or patient consent. The construction of each type of case is outlined below.

#### 3.6.1. Case Model

A case model is an example that illustrates all the concept’s attributes [28]. Mr. J. is an 83-year-old man, who lives with his wife of the same age in a village. Both are practicing Catholics. He has two children who emigrated to France. He visits them for a month in the summer. He has a family (nephews and brothers) who live nearby and provide support. Although Mr. J. suffers from several comorbidities associated with a chronic disease, he remains autonomous in his daily activities.

During the 2nd wave of COVID-19, Mr. J. presents an acute myocardial infarction and is accompanied by the emergency medical team to the emergency room of the nearby hospital. His children are informed but do not consider the possibility of death. On admission to the hospital, he tested negative for SARS-CoV-2. Clinical stability is achieved, and he is conscious and oriented. Due to restrictions, face-to-face visits are not allowed, but the family (niece and wife) talk to him by phone. Mr. J. does not understand the family’s absence and expresses concern for his wife who is alone at home. 

Due to the lack of differentiated care, he is transferred to a central hospital to perform cardiac catheterization. This takes place without incident, and he returns to the previous hospital. As there are no complications, a discharge plan is formulated. The day before discharge, he began to present a fever and worsened respiratory symptoms, suggestive of COVID-19, which was confirmed. Following a sudden and rampant worsening, he is transferred to the Intensive Care Unit (ICU). His clinical situation continues to decline, and the family is contacted to say goodbye. 

The niece contacts the children, who are incredulous and displeased at the lack of timely information about the patient. They seek a virtual visit, which is refused due to a lack of resources. They try to come from France that day but find it very difficult to leave the country. The wife says she is praying for the improvement of her husband’s condition and refuses to visit for fear of catching COVID, as she also has several comorbidities. The niece visits Mr. J. but finds him sedated and ventilated and feels she can no longer communicate with him. The niece is disturbed by the scenario, showing uncontrollable crying and marked anxiety, “But he’s already dead... what do I do now!” [sic]. The visit is carried out with all the protective equipment, but the niece, when prevented from touching her uncle, showed intense feelings of self-blame (sadness, anguish, helplessness, distress, remorse, etc.). 

That same night, Mr. J. died without the possibility of receiving a visit from his children, or religious support. The information is transmitted by telephone, during which the professionals express regret for the difficulty in communication and guarantee that Mr. J. and his relatives’ wishes will be satisfied.

Three days after the death, the funeral takes place without the usual farewells and funeral rites or the possibility of significant persons contacting the corpse (impossibility of an open coffin). The wife finds it difficult to accept that Mr. J. has died: “I don’t even know if it’s my husband who is in the coffin, they gave me back the clothes I sent to the funeral home... this is not dignified!” [sic]. 

The widow is left without close family support. Over time, and due to the traumatic loss, she exhibits disruptive behavior, manifested by daily visits to the cemetery, neglecting self-care, disbelief, and negative thoughts about the future. 

The widow often expresses the desire to be comforted by the people of her community, justifying her absence from the community by the village’s installed fear that the whole family could be infected by SARS-CoV-2 (false belief). 

This case is a model of being unprepared for death and represents the vast majority of the attributes that characterize the concept [28].

#### 3.6.2. Borderline Case 

A borderline case is similar to the concept of interest but does not include all its defining attributes [28]. Mr. C. is a 41-year-old married salesman with a three-year-old son. He goes on a business trip to Milan in early March 2020 and, one week after his return, he presents a high fever and acute respiratory distress. He waits a week at home, but given his worsening symptoms, he is taken to a central hospital, admitted to the pulmonology service, and subsequently diagnosed with COVID-19. Given the risk of transmission, isolation measures, and visitation restrictions are initiated. Given the sudden and threatening character of the disease, Mr. C. manifests multiple family, financial, and professional concerns. 

Already manifesting tiredness, he calls his wife by cell phone and says, “I will be fine, for you and our boy” [sic]. He also refers to concerns about the payment of taxes and access codes to accounts, providing his wife with guidance. After saying goodbye, he cries copiously and expresses to the nurse that he does not want to leave his family with problems to solve. He adds, “I’m not ready to die and I hope I don’t die like this” [sic]. Mr. C.’s health condition worsens, and it is necessary to intubate and sedate him. Given the service’s heavy workload, the family is not informed immediately. A day after being admitted to the Intensive Care Unit (ICU), Mr. C. dies. He was unable to say goodbye to his family as he wished. The family is contacted and informed about Mr. C.’s sudden death and that, due to the State of Emergency, no funeral rites can be performed. The family reacts to this news with dismay. The healthcare professional justifies the lack of previous contact by referring to the virus’ unknown behavior and the constraints associated with the increasing number of infections. 

This is the borderline case, highlighting about 50% of the attributes [28] of non-preparedness: unpredictability and sudden character; separation distress; poor quality of communication between family and professionals; lack of team strategies; and absence of rituals. However, Mr. C. is aware of his condition, tries to make plans, and communicates his wishes and concerns to family and professionals.

#### 3.6.3. Contrary Case

According to Walker and Avant, a contrary example is a case that does not exemplify the concept under analysis [28]. Mrs. M. is an autonomous elderly woman with comorbidities, living in a nursing home, and having family support from her three children. In March 2021, she began to have respiratory distress compatible with a moderate SARS-CoV-2 infection. Tests confirm she has COVID-19, and she is admitted to a hospital unit. During hospitalization, she is accompanied by a multidisciplinary team. The course of the disease is irregular, and in periods of greater stability, she manifests a desire to return home. The team is sensitive to these wishes and family members are mobilized to plan a possible return home. Given the restrictions during the pandemic’s 2nd wave, family members communicate with Mrs. M. via video call. In one of these conversations, she expresses her willingness to confess and speak openly about her death. The medical team convenes a family conference to discuss advanced care planning. She verbalizes: “I don’t want to be a burden for my family... I know that death is coming, and I don’t want to suffer.” [sic]. Given this manifestation, she returns home, thus respecting Mrs. M.’s preferences. The minimum safety conditions are ensured, with the agreement of the care teams and family. The home team stipulates pleasurable activities and ensures family visits through the bedroom window (since it is on the ground floor) with the use of intercoms. Faced with a worsening condition, Mrs. M. refuses to return to the hospital. This information is shared with her children. Mrs. M. asks to see her grandchildren through the window. She shares her decisions with her children, says goodbye, and reviews guidelines about her end-of-life wishes.

This case refers to the opposite model, including the following attributes regarding the preparation for death: awareness and preparation for death through interactions between the patient, family, and health team, as well as acceptance of death with a plan for the dying process.

### 3.7. Operative Definition 

The concept of death unpreparedness is defined as a transition in which the processes of information, communication, and relationship between the dying person, family, and HCP are compromised. This process anticipates a lack of awareness about preparing for death and difficulty accepting the loss. Often the dying person and their family are not included in the EoL decision-making process. The key components of the concept include uncertainty and unpredictability given the virus’ unknown behavior, the rapid decline generating separation distress, and the incapacity to fulfill wishes and death preferences, determining changes to farewell dying rituals. This transition often evolves in two opposite ways: (1) post-traumatic growth and death acceptance, or (2) a misadjusted loss process with a high risk of prolonged grief.

**Figure 2:**
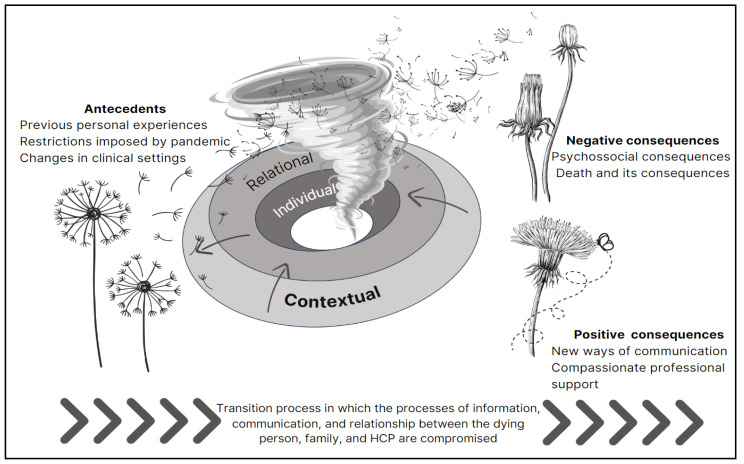
Conceptual representation of death unpreparedness due to a pandemic scenario.

The constant use of military metaphors and harsh language in medicine should be discarded since they fundamentally reinforce fear, stigma, and anxiety [67]. Given this effect and because metaphors are pervasive in human cognition and behavior, we picked an ecological metaphor to emphasize the caring, healing, and humanizing dimensions of healthcare. For better understanding, we use the dandelion symbolism to represent the concept of death unpreparedness (see Figure 2). Dandelions are renowned for their capacity to flourish in difficult environments. Like dandelions, human beings can access their inner fortitude, endure life’s trials, and overcome adversity. In various cultures, dandelions are a potent symbol of fortitude, hope, determination, and the capacity to surmount challenges due to their remarkable resilience. However, when bioecological domains (personal, relational, or contextual factors) behave as obstacles, some negative consequences emerge, particularly related to worse bereavement outcomes. After a sudden “hurricane”, like a pandemic, some dandelion seeds collapse, but others survive, land, and grow, typically when the environmental conditions are favorable (e.g., support of others). By embracing the dandelion’s symbolism, we can develop an appreciation for these tenacious blossoms and their potent bioecological message of fortitude in the face of adversity, such as a pandemic.

## 4. Discussion

This concept analysis demonstrates that the unpreparedness for death during COVID-19 is conditioned by individual, relational, and contextual aspects. Non-preparedness for death is related to a state in which an individual or a community is not ready psychologically, emotionally, or spiritually to deal with death and loss. Before COVID-19, this concept was coped with differently [26]. COVID-19 has drastically changed daily life in different ways, exposing major weaknesses around the world [68,69] and generating uncertainties in patients, families, caregivers, professionals, and the community itself. This implied changes in how they perceive, experience, and prepare for death and dying [70,71]. 

Before COVID-19, death preparedness was conceptualized as a transition process facilitated by communication with a healthcare provider, which leads to awareness and/or acceptance of end-of-life, as evidenced by the implementation of a plan [19]. After COVID-19, the measures quickly imposed to face the disease’s uncertainty constrained communication between patients/families and healthcare professionals and made it impossible to implement a plan, thereby changing the concept of death preparedness [72]. 

The concept of uncertainty in illness refers to the cognitive processes through which individuals interpret events associated with illness and derive meaning from them. This concept is characterized by the inability to ascertain the significance of illness-related events, which occurs when decision-makers are unable to definitively assign value to objects or events, or accurately predict outcomes [73,74]. This theory encompasses information obtained from the field of nursing as well as other disciplines. It focuses on clinical phenomena seen in clinical settings and aims to explain the process of assigning meaning to the experience of illness [73]. This lack of preparedness can impose negative consequences for people who are dying and their family/friends who have to deal with EoL issues and with loss and grief.

Not being prepared for death is a potential problem for all persons involved, implying psychosocial consequences, such as stress, anxiety, depression, and fear [33,46,50], and the consequences of death, such as prolonged grief symptoms [33,47,55]. The evidence indicates new ways of communication, compassionate professional support, and effective remote communication [40,47,51,52,53,55,57,64].

Enhancing familial relationships is unlikely to significantly extend a patient’s lifespan, but it may enhance their overall quality of life. Improving communication within a pandemic might potentially alleviate the emotional strain experienced by both impacted families and healthcare professionals. It is essential to encourage open and compassionate conversations about EoL matters, to provide support in coping with the emotional and spiritual aspects of dying, and to guarantee that patients’ wishes are respected.

All the experience gathered about death and dying with COVID-19 should be a lesson for the future, of health professionals, health managers, families, informal caregivers, and the general community. 

### 4.1. Study Limitations

A limitation of using Walker and Avant’s approach [28] is the absence of a specific strategy suggested for delineating the applications of the concept. Nevertheless, the use of a comprehensive literature review in our study mitigated this constraint. 

There are diverse cultural aspects surrounding death and dying, and different ways to grieve around the world. This makes it difficult to describe a normalized idea about attitudes and behaviors regarding bereavement. By excluding articles written in Chinese that refer to experience on the topic, we excluded experiences from the epicenter of COVID-19. Only a few of the available articles were focused on affected people. Furthermore, we did not assess the methodologic quality of the articles (i.e., heterogenous samples; incomplete information, and low evidence level) nor did we estimate interrater reliability using the kappa statistic.

Notwithstanding these limitations, the present analysis can help develop a wide language that encompasses the concept. By basing the constructed cases in this analysis on real-world scenarios, a greater degree of realism was achieved.

### 4.2. Implications for Practice

HCPs have a crucial and significant role in delivering both health and psychological care during a pandemic. Enhancing communication and involving families in care choices are crucial strategies for mitigating the trauma often associated with death experiences [32,47,55]. The intent is to ensure the secure organization of family visits in healthcare settings. In cases where physical visits are not feasible, alternative methods of remote communication should be employed to connect families and loved ones. These communication methods should also support collaborative efforts among healthcare teams, families, and patients [40,44,63]. 

Despite restrictions, HCPs should explore families’ ways of finding meaningful connections and of saying goodbye [40,48], promoting active family engagement to advocate wishes/preferences, and attending unfinished business [3,55,57]. 

Additional investment is required to enhance the availability of customized bereavement support services to effectively address the varied needs and backgrounds of those who have experienced loss. This includes the provision of culturally sensitive and crisis/contextually competent care, as well as the implementation of group-based support programs that cater to individuals with common experiences and traits. To proactively engage communities and increase knowledge of available support choices, it is recommended that information on grief and bereavement services be provided [45,75,76]. 

The promotion of self-care and reflection, along with the provision of psychological support, is crucial within healthcare teams [3]. To address this need, we recommend the development of training programs focused on mental health promotion and resilience skills [41]. 

## 5. Conclusions

In summary, COVID-19 brought new challenges for emotional, physical, and spiritual resources affecting the preparedness for death. Death unpreparedness emerged as a transition and a process characterized by uncertainty and unpredictability throughout the disease, generating separation distress and incapacity to manage existential issues to assure a good death. This transition may manifest in two contrasting outcomes: (1) post-traumatic development and acceptance of death, or (2) a maladaptive grieving process with a heightened susceptibility to extended suffering. It is essential to embrace strategies capable of providing emotional and spiritual support in the dying process and respect patients’ wishes, namely in catastrophic scenarios. In this sense, disaster management training will equip HCPs to discuss EoL priorities and the dying process with patients suddenly affected by a life-threatening infectious illness; to inform patients’ families of their deaths; and to ensure patients are well-informed about the EoL, if applicable. Lessons learned from COVID-19 should be present in similar events with a comparable impact to minimize their consequences.

## Figures and Tables

**Table 1:** Concept-defining attributes emerged as clusters of differentiating characteristics, from the perspective of the individual, relational, and contextual spheres.

Attributes		Dying Person	Family	Healthcare Professionals
**Individual**	**Disease-related factors**	- Unexpected [32,33] and sudden death [34]; - Unknown behavior of the virus, rapid decline [35].	- Cause of death and time since the loss [36]; - The unexpectedness of death [37]; the sudden death of relatives and friends [38].	N/A
	**Separation distress**	N/A	- Distress and fear of the unknown and trajectory of illness [39]; about the future [34] - Emotional shock: (i) due to uncertainty [34,40]; (ii) from the sudden death of a family member/relative/friend) [34,36,38]; - Feeling traumatized, conflicts with the deceased [33], and inability to say goodbye before, during, and after death [41,42]; - Despair, anguish, and stress that the patient died alone [32]; - Self-blaming emotions (regrets, guilt, shame, and anger as loos-oriented stressors) [43,44]; - Non-acceptance of death grief or bereavement support [44,45].	N/A
	**Scarcity of death and grief literacy**	N/A	- Lack of literacy about death and grief (discomfort asking for help and how to access services) [45]; - Caregiving unpreparedness [36,46];	N/A
**Relational**	**Dying alone**	- Dying without ceremony [46,47] - Lack of presence before death or family or health care support [46,47] - Lack of supportive and compassionate care [45]	- Feeling unsupported by health care professionals) [48]; - Anxiety-inducing loneliness accompanied by profound depression and the imperative to protect the integrity of interpersonal connections [49]; - Disrupted process of saying goodbye [40,44].	N/A
	**Poor interpersonal communication**	N/A	- Poor quality communication [32,40,42,43,50,51]; - Absence of strategies for remote communication or poor virtual communication [40,52].	- Lack of proactive communication from HCPs [47]; - Lack of attentiveness and Communication [53]; - Insufficient communication concerning the patient’s condition, and failure to involve the family member in the decision-making process regarding care [32]; - Lack of strategies based on communication technologies [54]; - Communication without death rituals [32,40].
	**Transcendence facing existential issues**	- Unfinished business [43]	- Dichotomy hope and despair [35]; - Prayer as a resilience factor and stress restoration [49]; - Unfinished business—secrets [43].	- Unfacilitated active family engagement to advocate wishes/preferences evident [55]; - Family caregivers fear of the medical staff [35].
**Contextual**	**Disrupted collective mourning and grieving**	N/A	- Loss of significance of the funeral rituals [41]; - Lack of funeral rituality—a proper funeral [43,49]; - Disrupted collective mourning and grieving [45].	N/A
	**Disrupted compassionate care**	- Non-individualized care [7]	- The impossible direct presence of family at the moment of death [43,50,56,57]; - “Stolen moments” after the patient’s death and feelings of disbelief [50]; - Unclear decision-making [43]; - The imagery of the loved one “struggling for life on some machine” hints at the impersonal and traumatic impact of COVID-19 deaths [57]; - Non-delivery of compassionate care (lack of regard for meeting families); emotional needs [55].	N/A
	**Pandemic Social Stigma**	- Dying without religious ceremony [9]; - Social stigma [9].	- Unsafe, coexisting with other stressors [40]; - Struggled to contain the spreading of the virus into the family [40].	N/A

N/A: Not Applicable.

**Table 2:** Consequences of death unpreparedness due to COVID-19.

	Bereaved Family	Healthcare Professionals
**Negative**	**Psychosocial consequences** Emotional dysregulation, e.g., feelings of guilt, rumination [34,39,55,63] unreality, and powerlessness [50] Impact on mental health status, e.g., stress, depression, and anxiety symptoms [33,46] Fear of the future, e.g., lack of job and financial security [34] Instability in the family and social interactions [34,48] **Death and its consequences** Changing farewell rituals [34,36,39,40,42,44,50,60,63,64] Damaging effects on grief [33,43,55,57] Prolonged grief symptoms [33,47,55] Bereavement overload [60] Death in loneliness [50,63,64] Unnatural Mourning [39,42] Loss of carer role [39] Difficulties in establishing rapport and bonding with the medical team [50] Ambivalence—Technology available but not desired [32,47,50] Lack of bereavement or mental health services [45] Institutionalized death, a higher risk of poor experiences in end-of-life compared to death in place (home) [60]	Self-perception of anguish [65] Low quality of life [65]
**Positive**	New ways of communication—technologically mediated contact, e.g., telephone; virtual healthcare team–family communication [3,40,51,57,64] Compassionate professional support [53,55] A significant correlation was observed between effective remote communication with the patient and the health care team and improved family experiences during EoL care [47,52]	

## Data Availability

All data generated or analyzed during this study are included in this article.

## Supplementary Materials

The following supporting information can be downloaded at: https://www.mdpi.com/article/10.3390/healthcare12020188/s1, Table S1. Characteristics of the studies included in the review (n = 34).
