# Supplementary material for: Death Unpreparedness Due to the COVID-19 Pandemic: A Concept Analysis
Source: Healthcare (Basel). 2024 Jan 12;12(2):188. doi: 10.3390/healthcare12020188 (PMC10815185; doi:10.3390/healthcare12020188)
Supplement: Supplementary file 1 [file healthcare-12-00188-s001.zip › healthcare-2721775-SI.pdf]

Table S1. Characteristics of the studies included in the review (n=34).

Author(s)/ Year / Country	Type of Study	Aims	Setting/ Participants	Key findings
Feder et al. (2021) [32]<br><br>USA	Qualitative study	To examine bereaved families' perceptions of the quality of end-of-life communication among veterans, families, and staff in Veterans Affairs (VA) medical centers during the COVID-19 pandemic.	VA medical centers with the highest numbers of COVID-19 cases during the study period. Next-of-kin of 328 Veterans who died in one of 37 VA medical centers' acute care, intensive care, nursing home, or hospice units.	Communication between "patients, families, and healthcare teams at the EoL remains critically important during times of limited in-person visitation. Families reported that low-quality communication causes profound distress that can affect the quality of dying and bereavement" (p.587).
Tang et al. (2021) [33]<br><br>China	Descriptive-correlational study	To assess the mental health of Chinese adults bereaved by COVID-19 and analyze the associations of demographic and loss-related characteristics with mental health after COVID-19 bereavement.	Adult participants bereaved by COVID-19 (n=422) were recruited via advertisements on social network websites.	Several participants bereaved by COVID-19 experienced severe mental health problems. The recent loss of first-degree relatives, feeling traumatized by the loss, and having a close and/or conflictual relationship with the deceased may elevate the risk for these mental health problems, which could require psychological treatment.
Mohammadi et al. (2021) [34]<br><br>Iran	Qualitative study	To identify the Mental Health crises that the families of COVID-19 deceased victims are going through.	16 members of families that had lost a member to COVID-19 were selected via purposeful sampling.	The families of COVID-19 deceased victims were affected by various psychological crises that exposed them to a deep sense of loss and emotional shock. Therefore, there is an urgent need for a cultural context that recognizes and supports all the various aspects of these families' mental health.
Rababa et al. (2020) [35]<br><br>Jordan	Descriptive quantitative study	To examine the association of death anxiety with religious coping and spiritual well-being among 248 community-dwelling older adults during the COVID-19 pandemic.	248 community-dwelling older adults aged between 60 to 75 years.	Most of the participants exhibited low levels of religious coping and spiritual well-being and high levels of death anxiety in comparison to male older adults; female older adults had higher levels of religious coping and lower levels of death anxiety in comparison to widowed older adults; married older adults had higher levels of death anxiety.
Eisma & Tamminga (2022) [36]	Comparative study	To compare loss characteristics, loss circumstances, and grief levels among people bereaved by	Adults who experienced deaths due to COVID-19 (n=99), natural causes (n=1006) and unnatural (n=161) causes were recruited	Grief due to COVID-19 is characterized by a unique set of loss characteristics and circumstances and elevated grief levels. Improving opportunities to say goodbye before and after death may provide an important means to

Netherlands		COVID-19 and by natural and unnatural causes.	online through a Dutch national mental health care organization’s website.	prevent and reduce severe grief following COVID-19 deaths.
Eisma et al. (2021) [37]<br><br>Netherlands	Descriptive-correlational study	To compare grief levels among people recently bereaved by COVID-19 and by natural and unnatural causes.	Adults bereaved due to COVID-19 ($n = 49$), natural causes ($n = 1182$), and unnatural causes ($n = 210$) were recruited via advertisements on social network websites and mobile applications.	Higher grief levels occurred among people bereaved by COVID-19 compared to people bereaved by natural loss. We predict that pandemic-related increases in pathological grief will become a worldwide public health concern.
Wang et al. (2022) [38]<br><br>USA	Descriptive quantitative study	To examine the association between COVID-19 bereavement and depression reported by older adults in 27 countries and test for variations by gender and country.	51,383 older adults (age 50–104) living in 27 European countries of whom 1,363 reported the death of a relative or friend from COVID-19.	Surviving elders experienced enduring mental health consequences as a result of COVID-19 fatalities. Despite the evident impact of the crisis, elderly folks who lost loved ones were especially in need of mental health support.
Hack et al. (2021) [39]<br><br>Australia	Mixed methods study	To explore end-of-life experiences of residents who died in residential aged care facilities and of their next-of-kin/carers during the COVID-19 pandemic; to identify areas of concern and areas for improvement.	Participants were next-of-kin or carers of residents referred to Hospital-based Residential InReach teams, and who died within 30 days of referral, during the ‘second wave’ of COVID-19.	Five major themes were identified: (i) COVID-19 pandemic; (ii) communication and technology; (iii) death and dying; (iv) bereavement and grief; and (v) social supports and external systems. Constraints were also identified in the access to palliative care and bereavement support for dying residents and for grieving carers due to the pandemic.
Hanna et al. (2021) [40]<br><br>UK	Descriptive qualitative study	To explore health and social care professionals’ experiences of providing EoL care during the COVID-19 pandemic to help inform current/future clinical practice and policy.	Sixteen health and social care professionals working across a range of clinical settings (hospital, hospice, and care home) by supporting dying patients during the first wave of the COVID-19 pandemic.	Participants reported “emotional and practical challenges in providing EoL care during the pandemic, including increases in patient numbers, reduced staffing levels, and reliance on virtual platforms for sensitive, emotive conversations with relatives. Participants were central to promoting connections between patients and their families at EoL and creating opportunities for final contact before death. However, providing support varied because of the pandemic’s pressures. Results are discussed under two themes: (1) challenges and facilitators to providing EoL care, and (2) support needs of relatives when a family member was dying during the COVID-19 pandemic” (p.1249).

Hernández-Fernández & Meneses-Falcón (2021) [41]<br><br>Spain	Interpretive qualitative study	To analyze the experience of losing a loved one without traditional, culturally specific rituals for saying goodbye; explore the different factors affecting the onset of mourning by family members; and study the existence of complicating risk factors associated with grief from this distinct type of loss.	48 informants (mourners and professionals who were direct witnesses to the deaths) during the COVID-19 pandemic, in Madrid.	(a) due to their characteristics, deaths caused by the pandemic were a complicating factor for bereavement; (b) professionals who supported these deaths with a holistic approach facilitated the process for the family members, enabling the beginning of the mourning process and reducing anguish for the family members; and (c) the funeral rite is in need of a resignification.
Selman et al. (2021) [42]<br><br>UK	Descriptive quantitative study	To explore British newspaper representations of 'saying goodbye' before and after a COVID-related death and consider clinical implications.	711 participants were recruited via media, social media, national associations, and other organizations.	The act of 'saying goodbye' (before, during, and after death) was central to the media's representation of COVID-19 bereavement;<br>Bedside access was portrayed as restricted, variable, and uncertain, with families begging or bargaining for contact;<br>Patients were portrayed as 'dying alone' regardless of the clinician's presence;<br>Funerals were portrayed as travesties and grieving alone as unnatural. Articles focused on what was forbidden and offered little practical guidance.
Lee et al. (2022) [43]<br><br>(USA)	Descriptive quantitative study	To study grief experiences or mourners who lost a loved one to COVID-19 with a focus on self-blaming emotions and unresolved issues with the deceased.	209 American participants bereaved by COVID-19.	"Universal endorsement of one or more forms of self-blame (guilt, regret, shame) or unfinished business (UB), with over one-third of mourners endorsing all four experiences. Those with a closer relationship with the deceased reported both greater distress over unfinished business and more intense and dysfunctional grief symptomatology. Strikingly, unresolved conflict (a major dimension of UB) accounted for nearly 40% of the unique variance in problematic grief, yet was unrelated to time since the loss" (p.1297).
Torrens-Burton et al. (2022) [44]<br><br>UK	Descriptive qualitative study	To analyze qualitative data from two independent UK-wide online surveys describing the experiences of 881 people bereaved during the pandemic.	881 people bereaved during the pandemic in the UK.	"Identified six main themes: troubled deaths; mourning, memorialization and death administration; mass bereavement, the media and the ongoing threat of the pandemic; grieving and coping; work and employment; and support from the health and social care system" (p.1).

				Loss-oriented stressors included being unable to visit and say goodbye at the EoL, and restricted funeral and memorialization practices. Associated reactions were feelings of guilt and anger, and problems accepting the death and beginning to grieve.<br>Examples of restoration-oriented stressors and reactions were severely curtailed support-systems and social/recreational activities, which impacted people's ability to cope.
Harrop et al. (2021) [45]<br><br>UK	Quantitative data from a longitudinal survey	To investigate grief experiences, support needs, and use of formal and informal bereavement support among people bereaved during the pandemic.	711 adults bereaved in the UK, recruited via media, social media, national associations and community/charitable organizations.	People bereaved during the pandemic had high levels of support-needs, alongside difficulties accessing support.
Breen et al. (2021) [46]<br><br>USA	Descriptive quantitative study	To determine how psychological symptoms explain functional impairment.	People bereaved by COVID-19 (n=307) in the USA.	Odds of functional impairment significantly increased 27% for higher scores in separation distress, 25% for higher scores in dysfunctional grief, and 13% for higher scores in post-traumatic stress;<br>People bereaved because of COVID-19 are at risk of functional impairment, especially if they have symptoms of separation distress, dysfunctional grief, and/or post-traumatic stress.
Schloesser et al. (2021) [47]<br><br>Germany	Descriptive quantitative study	To describe the experiences of bereaved relatives of patients who died during the SARS-CoV2 pandemic, regardless of whether patients were infected with SARS-CoV2 or not.	81 bereaved relatives of people who died during the pandemic in Germany, with and without SARS-CoV2 diagnosis.	Relatives of COVID-19 patients felt burdened by the visiting restrictions and they suffered from pandemic-related stress;<br>Patients died alone due to visiting restrictions;<br>The burden for relatives in the hospital setting was higher compared to relatives of patients who died at home.
Selman et al. (2022) [48]<br><br>UK	Descriptive quantitative study	To identify clinical and demographic risk factors for sub-optimal EoL care and pandemic-related challenges before death and in early bereavement; to inform clinical	711 participants were recruited via media, social media, national associations and organizations.	Four risk factors were found for poorer end-of-life care and pandemic-related challenges in bereavement: place, cause; death expectedness; and relationship to the deceased.

		practice, policy and bereavement support.		
Testoni et al. (2021) [49]<br><br>Italy	Interpretative qualitative study	To investigate the psychological experiences related to the contagion and the eventual death of colleagues as well as the resilience strategies activated by priests during the process.	12 catholic priests from the same pastoral community in one region in Northern Italy that was most affected during the 1st phase of the pandemic. Ministers have been in constant contact with the faithful of their parishes since the breakout of the virus.	The areas studied concerned the experiences of the participants during the lockdown, the implications of social distancing and lack of funeral rituality, and the importance of prayer as a resilience factor.
Kentish-Barnes et al. (2021) [50]<br><br>France	Descriptive qualitative study	To understand the experiences of bereaved family members of patients who died in an ICU during the COVID-19 pandemic, from the time of hospital admission until after the patient's death.	19 bereaved family members of patients who died from severe COVID-19 in 12 ICUs during the first wave of the pandemic in France	During the initial wave of the COVID-19 pandemic in France, bereaved family members described a disturbed experience, both during the ICU stay and after the patient's death. Specific family-centered crisis guidelines are needed to improve experiences for patients, families, and clinicians' experiences.
Lawlor et al. (2022) [51]<br><br>Canada	Comparative study	To compare EoL in terms of in-person family presence, patient–family communication, and healthcare team–family communication in hospitalized decedents before and during the COVID-19 pandemic.	One quaternary and two tertiary adult acute care hospitals in Ottawa, Canada.<br>Pre-COVID-19 pandemic decedents (n=892) and decedents during the COVID-19 pandemic's first wave (n=1142).	In "hospitalized COVID-19 pandemic wave 1 decedent, in-person family presence and in-person team–family communication encounters decreased at EoL; virtual modalities were adopted for communication, and telephone use increased in team–family communication encounters" (p.8).
Ersek et al. (2021) [52]<br><br>USA	Mixed methods study	To examine the impact of remote communication on families' evaluation of EoL care during the COVID-19 pandemic.	Next-of-kin of 328 veterans who died in one of 37 Veterans Affairs medical centers' acute care, intensive care, nursing home, or hospice units.	Effective remote communication with the patient and the health care team was associated with significantly better ratings of the overall experience of EOL care by bereaved family members.
Becqué et al. (2021) [53]<br><br>Netherlands	Descriptive qualitative study	To provide insight into aspects of EoL care practices that might have jeopardized or supported the dignity of patients and their family members during the	25 bereaved relatives of patients who died during the COVID-19 pandemic.	Most impacts on the dignity experiences of relatives were based on human actions and relationships.<br>Relatives experienced that preventive measures could be mitigated by healthcare professionals to make them less devastating.

		first wave of the COVID- 19 pandemic.		
Romero et al. (2022) [54]<br><br>Spain	Descriptive qualitative study	To describe how the COVID-19 pandemic affected nursing homes and primary care professionals' attempts to achieve the objectives of a pre-existing EoL program and to explore their personal experiences of EoL care in these facilities.	20 professionals from nursing homes and primary care facilities who participated in NUHELP program development.	The pandemic cast light on existing shortcomings in nursing homes in terms of comprehensive assessments, communication, decision-making, grief management, and palliative care complexity. Nursing homes need more human, material, and training resources, as well as improved coordination with the public healthcare system.
Mayland et al. (2021) [55]<br><br>UK	Observational study	To explore bereaved relatives' experiences of the quality of care and family support provided during the last days of life; to identify the impact of factors associated with perceived support.	278 adult people who had experienced the death of a relative/friend (in all care settings).	Despite public health restrictions, "individualized care can be enabled by proactive, informative communication; recognizing dying in a timely manner and facilitating the ability to be present before death" (p.1480).
Ham et al. (2021) [56]<br><br>Netherlands	Observational study	To provide insight into the impact of the COVID-19 pandemic on the quality of life, social support, and self-care of bereaved relatives of people with advanced cancer; to evaluate whether care for bereaved relatives during the COVID-19 pandemic should be improved.	People with advanced cancer and their (bereaved) relatives (n=91).	In the short term, the COVID-19 pandemic did not have a significant impact on bereaved relatives' wellbeing. However, the long-term impact of the pandemic on their wellbeing should be assessed.
Neimeyer & Lee (2021) [57]<br><br>USA	Secondary analysis of e-survey	To examine the relationship between the reactions of the bereaved to pandemic conditions and the severity of their grief and levels of impairment.	831 American Adults, who lost a loved one due to COVID-19.	Circumstantial risk factors accounted for 59% of the variance in social impairment and 71% of the variance in pandemic grief, leading to the development of an inventory of Pandemic Grief Risk Factors (PGRF), which displayed a unified factor structure, high reliability, and strong convergent validity.
Ishikawa (2020) [58]	Narrative review	To discuss loss and grief processes among older adults	N/A	Older adults' "struggles with loneliness, fear of dying, and the sequelae of untreated medical conditions are viewed

USA		during the COVID-19 pandemic.		through the lens of anticipatory grief, and coping and treatment strategies are offered” (p.S85).
Firouzkouhi et al. (2022) [59]<br><br>Iran	Descriptive qualitative study	To explore the experiences of critically ill patients with COVID-19 about death and dying.	12 critically ill patients with COVID-19 admitted to the ICU in southeastern Iran.	Data analysis generated two main themes, including personal and non-personal elements (challenging thinking about death) and eight sub-themes. Thinking about the death of critically ill COVID-19 patients is a significant challenge that affects the patient's health and prolongs the treatment process, and should be treated carefully in the patient's treatment and care program.
Kokou-Kpolou et al. (2020) [60]<br><br>France	Narrative review	To analyze factors related to bereavement in the context of the COVID-19 pandemic.	N/A	(a) probable multiple deaths for families could lead to “bereavement overload”;<br>(b) the viral contagiousness of COVID-19 deprives families of assisting the dying person in their last days of life and performing ritual ceremonies;<br>(c) the social and relational restrictions due to the epidemic prohibit families from visiting their relatives at hospitals or intensive care units, which may elicit or exacerbate feelings of guilt;<br>(d) excessive and collective accumulation of deaths could deny recognition of each individual’s bereavement;<br>(e) COVID-19-related deaths need careful attention to manifestations of prolonged grief symptoms during assessment and treatment.
Rao & Kelemen, (2021) [61]<br><br>USA	Qualitative case study	To describe the death experiences of four patients in an urban academic medical center who received very different degrees of medical intervention; to examine the interventions of the interdisciplinary Palliative Care team.	Four patients cared for in an urban academic medical center	Palliative Care teams must adapt to the new landscape by creating best practices for ensuring adequate symptom control, modifying approaches for withdrawal of life-sustaining medical technologies, and gaining facility with communication through teleconferencing platforms to meet the challenge of alleviating suffering for people dying from COVID-19.
Mitchell et al., (2022) [62]<br><br>UK	Mixed methods study	To provide detailed insights and understanding into service changes and innovations in EOL care services provided by	559 valid responses were received from 387 community nurses, 156 general practitioners, and 16 'others'.	Over a third of respondents (n = 224; 40.8%) experienced changes in their team’s organization to provide EoL care in response to the COVID-19 pandemic. “Three qualitative themes were identified: COVID-19 as a catalyst for change

		UK primary care units during the first phase of the COVID-19 pandemic.		in primary palliative care; new opportunities for more responsive and technological ways of working; and pandemic factors that improved and strengthened interprofessional collaboration” (p.161).
Delor et al., (2021) [63]<br><br>Italy	Multimethod qualitative study	To explore the families’ experiences and needs collected during follow-up calls, and the role psychologists played in the calls.	The Clinical Psychology unit of an Italian hospital offered a bereavement follow-up call to such families (246 families were called over 3 months).	Six themes emerged: lack of death rituals, solitary, unexpected, unfair, unsafe, and coexisting stressors. Families’ reactions were perceived by psychologists as close to traumatic grief. Families’ needs ranged from finding alternative rituals to giving meaning and expressing different emotions. The psychologists played both a social-institutional and a psychological-human role in the calls.
Mortazavi et al., (2023) [64]<br><br>Iran	Descriptive qualitative study	To gain a deep understanding of the experience of mourning during the COVID-19 pandemic by exploring the experiences of survivors of the death of their loved ones.	Family members of people who died from coronavirus disease were identified from records provided by organizations supporting the surviving families (n=15).	During the COVID-19 pandemic, the inability to hold the usual ceremonies for mourning and receive the social support needed in this period, the relatives of the deceased encounter various conditions that disrupt the grieving process and may lead to the spread of unresolved grief in the future.
Ferreira et al. (2021) [65]<br><br>Brazil	Quantitative study	To estimate self-perception of anguish and low quality of life among healthcare professionals who cared for dying patients during the COVID-19 pandemic; to determine the characteristics of healthcare professionals and patients and EOL care.	Healthcare professionals who cared for dying patients (N=102).	Self-reported anguish was more frequent in physicians and when disagreement about EoL care occurred. Low quality of life was more frequent when health care professionals did not have time to talk to patients’ relatives and was less frequent when health care professionals agreed that medical care was enough.
